# Bulk assembly of organic metal halide nanotubes†
†Electronic supplementary information (ESI) available. CCDC 1550500. For ESI and crystallographic data in CIF or other electronic format see DOI: 10.1039/c7sc03675b


**DOI:** 10.1039/c7sc03675b

**Published:** 2017-10-16

**Authors:** Haoran Lin, Chenkun Zhou, Yu Tian, Tiglet Besara, Jennifer Neu, Theo Siegrist, Yan Zhou, James Bullock, Kirk S. Schanze, Wenmei Ming, Mao-Hua Du, Biwu Ma

**Affiliations:** a Department of Chemical and Biomedical Engineering , FAMU-FSU College of Engineering , Tallahassee , Florida 32310 , USA . Email: bma@fsu.edu; b Materials Science and Engineering Program , Florida State University , Tallahassee , Florida 32306 , USA; c National High Magnetic Field Laboratory , Florida State University , Tallahassee , Florida 32310 , USA; d Department of Chemistry and Biochemistry , Florida State University , Tallahassee , Florida 32306 , USA; e Department of Chemistry , University of Florida , Gainesville , Florida 32611 , USA; f Materials Science & Technology Division and Center for Radiation Detection Materials and Systems , Oak Ridge National Laboratory , Oak Ridge , Tennessee 37831 , USA

## Abstract

The organic metal halide hybrids welcome a new member with a one-dimensional (1D) tubular structure. Herein we report the synthesis and characterization of a single crystalline bulk assembly of organic metal halide nanotubes, (C_6_H_13_N_4_)_3_Pb_2_Br_7_. In a metal halide nanotube, six face-sharing metal halide dimers (Pb_2_Br_9_^5–^) connect at the corners to form rings that extend in one dimension, of which the inside and outside surfaces are coated with protonated hexamethylenetetramine (HMTA) cations (C_6_H_13_N_4_^+^). This unique 1D tubular structure possesses highly localized electronic states with strong quantum confinement, resulting in the formation of self-trapped excitons that give strongly Stokes shifted broadband yellowish-white emission with a photoluminescence quantum efficiency (PLQE) of ∼7%. Having realized single crystalline bulk assemblies of two-dimensional (2D) wells, 1D wires, and now 1D tubes using organic metal halide hybrids, our work significantly advances the research on bulk assemblies of quantum-confined materials.

## Abstract 2

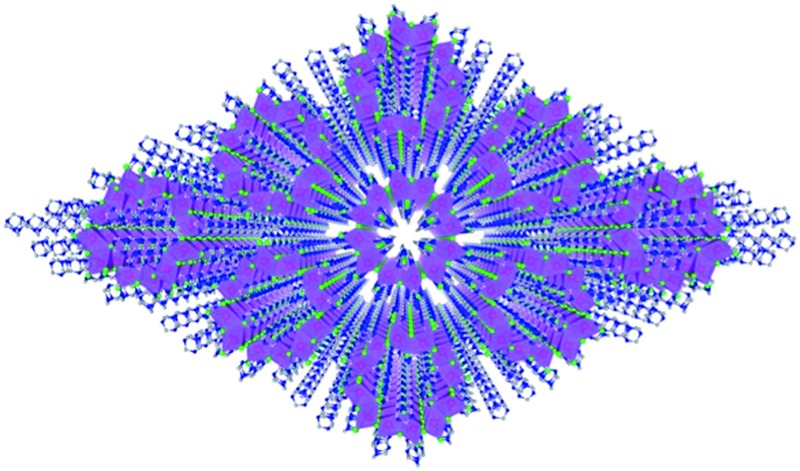
A single crystalline organic metal halide hybrid containing arrays of one-dimensional metal halide nanotubes is discovered for the first time.

## Introduction

Since the discovery of carbon nanotubes by Iijima in 1991,^1^ materials with tubular structures have attracted great scientific interest because of their intriguing physical and chemical properties. Besides carbon nanotubes, a number of synthetic tubular structures such as metal oxides, polymers, metal organic frameworks (MOFs) *etc.* have been developed over the last few decades,^2^–^8^ which show promising applications in various areas, ranging from gas separation and storage to catalysis and drug delivery. In spite of the advances in realizing tubular structures, it is still of great interest to develop discrete materials with functionalities as rich as those of carbon nanotubes for high performance devices.

Organic–inorganic metal halide hybrids have recently received tremendous research attention for their exceptional optical and electronic properties with useful applications in a variety of optoelectronic devices from photovoltaic cells to light emitting diodes, photodetectors, and lasers.^9^–^12^ The exceptional structural tunability of this class of materials enables the formation of various types of crystal structures by using appropriate organic and inorganic components, ranging from three- (3D), to two- (2D), one- (1D), and zero-dimensional (0D) structures on the molecular level.^13^,^14^ In the 3D structure, small size cations, such as Cs^+^ and CH_3_NH_3_^+^, fit into the corner-sharing metal halide octahedrons (MX_6_) to form a framework with the 3D MX_6_ network.^15^,^16^ In the 2D structure, the MX_6_ octahedrons are connected in layered or corrugated sheets at the corners.^17^–^19^ In the 1D structure, the MX_6_ octahedrons are connected in a chain^20^,^21^ and in the 0D structure, the MX_6_ octahedrons or assemblies of octahedrons are isolated from each other and surrounded by organic cations.^22^–^24^ These 2D, 1D, and 0D hybrids can be considered as bulk assemblies of core–shell quantum confined materials, with 2D wells, 1D wires, and 0D individual metal halide octahedrons confined by organic ligands. The isolation between the building blocks in these low dimensional metal halide hybrids enables the bulk materials to exhibit the intrinsic properties of the individual building blocks. In other words, organic metal halide hybrids enable the properties of conventional individual nanostructures to be present in single crystalline bulk assemblies with defined periodicities. The structural versatility of this class of hybrid materials offers a vast parameter space to explore novel crystal structures exhibiting diverse properties.

Herein, we report for the first time an organic metal halide hybrid with 1D tubular structure. By using a proper organic cation, protonated hexamethylenetetramine (HMTA) (C_6_H_13_N_4_^+^), we have been able to dictate the assembly of metal halides to form a bulk assembly of 1D metal halide nanotubes with a chemical formula of (C_6_H_13_N_4_)_3_Pb_2_Br_7_. Due to the complete isolation and strong quantum confinement of individual metal halide nanotubes by wide band gap organic moieties, this nanotube-assembled material (HMTA)_3_Pb_2_Br_7_ with 1D structure can exhibit the intrinsic properties of the individual metal halide nanotubes. A strongly Stokes shifted broadband yellowish-white emission peak at 580 nm with a large full width at half maximum (FWHM) of around 158 nm and a PLQE of around 7% was realized as a result of exciton self-trapping in the metal halide frameworks.^25^–^33^

## Results and discussion

**Fig. 1:**
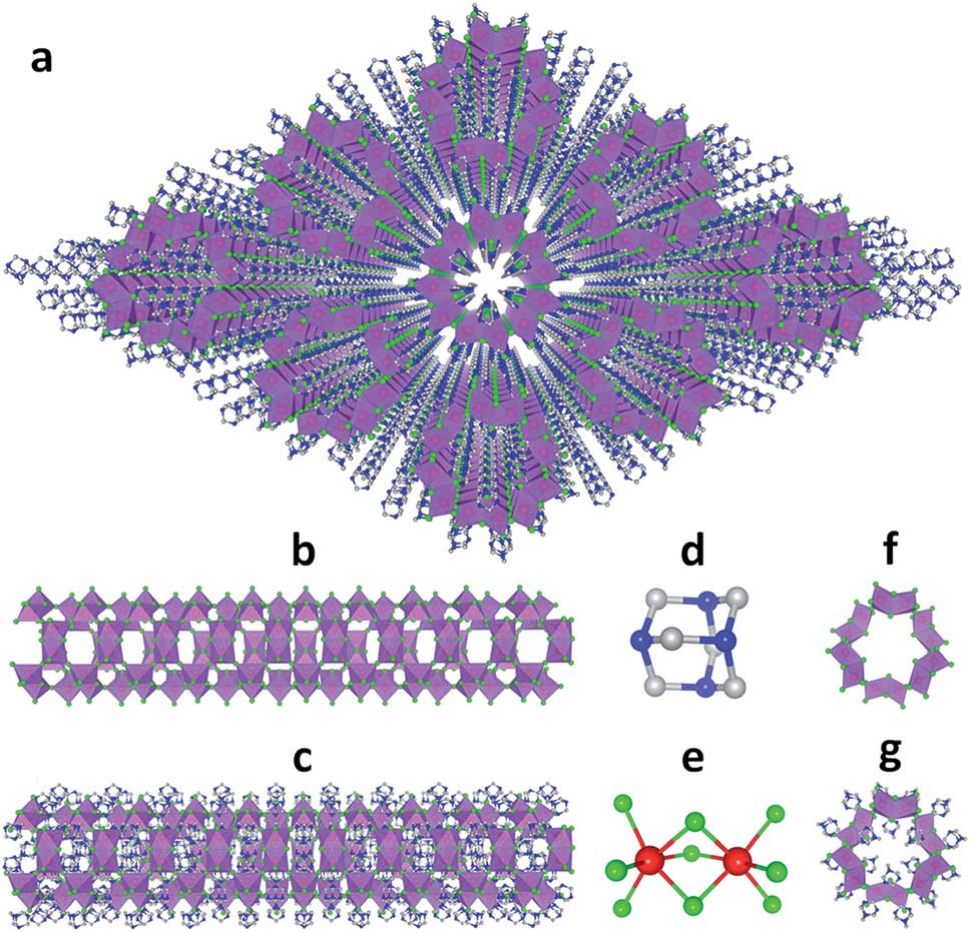
(a) View of the structure of (HMTA)_3_Pb_2_Br_7_ (red: lead atoms; green: bromine atoms; blue: nitrogen atoms; gray: carbon atoms; purple polyhedra: PbBr_6_ octahedra and Pb_2_Br_9_ dimers; hydrogen atoms are hidden for clarity). (b) Side view of an individual lead bromide nanotube. (c) Side view of an individual lead bromide nanotube surrounded by organic cations. (d) Ball-and-stick model of an individual HMTA unit (hydrogen atoms are omitted). (e) Ball-and-stick model of an individual face-sharing lead bromide dimer Pb_2_Br_9_ with detailed bond lengths and bond angles listed in the ESI.† (f) Top view of a rugged ring formed by six lead bromide dimers. (g) Top view of a rugged metal halide ring coated with organic cations inside and outside.

Single crystalline (HMTA)_3_Pb_2_Br_7_ was prepared by slow solution diffusion of dichloromethane into dimethylformamide (DMF) precursor solutions containing PbBr_2_ and hexamethylenetetramine hydrobromide (C_6_H_13_N_4_Br) (see experimental details in the ESI†). Single crystal X-ray diffraction (SCXRD) was used to characterize the structure (Tables S1 and S2†). Fig. 1a shows large arrays of organic metal halide nanotubes, with an individual stripped metal halide nanotube shown in Fig. 1b and surrounded by organic cations as shown in Fig. 1c. The two basic building blocks for this organic metal halide hybrid are protonated HMTA (C_6_H_13_N_4_^+^) (Fig. 1d) and face-sharing lead bromide dimers (Pb_2_Br_9_^5–^) (Fig. 1e). In an individual nanotube, six lead bromide dimers connect at the corners to form rugged rings (Fig. 1f), with an inner radius of around 4 Å, that extend one dimensionally. The varied Pb–Br bond lengths from 2.873 Å to 3.163 Å and Br–Pb–Br bond angles from 77.25° to 113.15° indicate that the present PbBr_6_ units are highly distorted and deviate from the typical octahedron configuration (Fig. S1, Tables S3 and S4†). In each of the “windows” formed by four connected dimers on an individual hollow nanotube, three protonated HMTA cations C_6_H_13_N_4_^+^ are anchored by coulombic interactions and hydrogen bonding, with one positioned inside the tube and the other two outside (Fig. 1g). In other words, individual negatively charged metal halide nanotubes are coated by positive organic cations on both the inside and the outside in a 1 : 2 ratio, resulting in an inorganic–organic core–shell nanotubular structure with an inner radius of around 1.5 Å when taking the organic cations into account. These organic coated metal halide nanotubes further assemble into a hexagonal close packed array in the macroscopic crystal (Fig. 1a). The powder X-ray diffraction (PXRD) pattern of the ball-milled sample is identical to the simulated PXRD pattern from the single crystal structure (Fig. S2†), proving the reliability of the SCXRD results. This hybrid material exhibited moderate thermal stability without decomposition up to around 155 °C in thermogravimetric analysis (TGA) (Fig. S3†). This organic metal halide hybrid with a unique nanotubular structure exhibits different properties from previously known organic metal halide hybrids, and could be of interest for various potential applications. Here we report its optical properties.

**Fig. 2:**
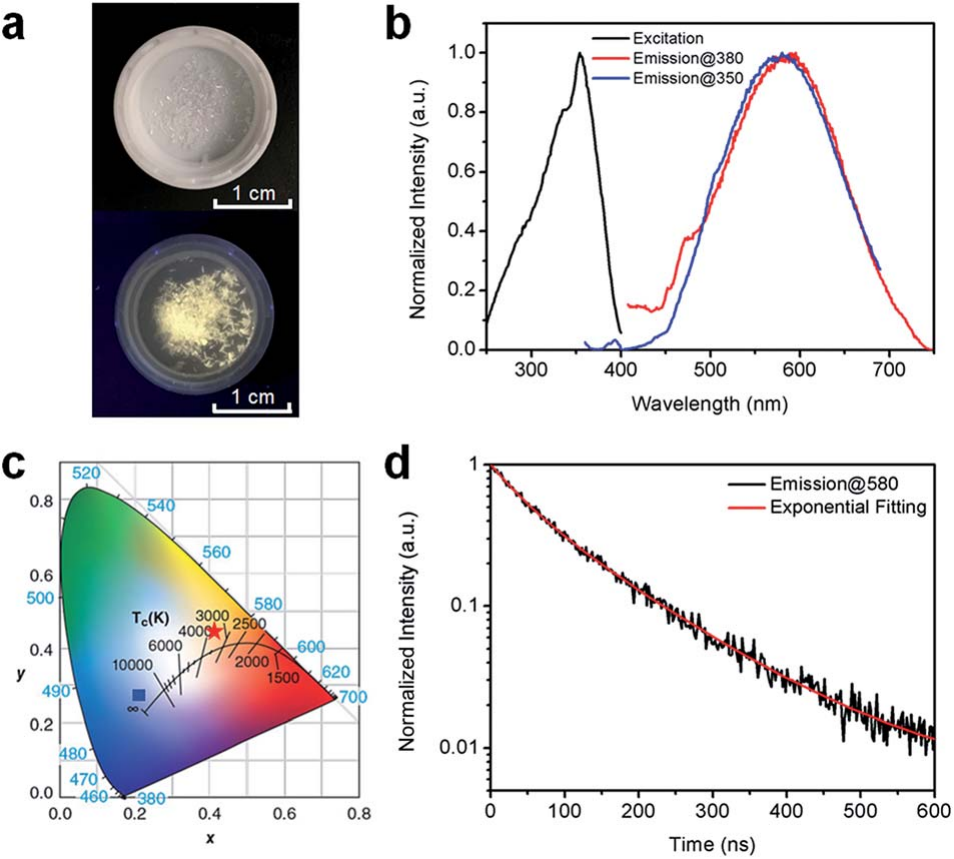
(a) Images of (HMTA)_3_Pb_2_Br_7_ crystals under ambient light (top) and UV light (365 nm, bottom). (b) Excitation (black line, probed at 580 nm) and emission (red line, excited at 380 nm; blue line, excited at 350 nm) spectra of (HMTA)_3_Pb_2_Br_7_ crystals at room temperature. (c) CIE chromaticity coordinates of the bulk assembly of 1D lead bromide nanotubes in this work (red star), and the bulk assembly of 1D nanowires (C_4_N_2_H_14_PbBr_4_) (blue square) (ref. 21). (d) Time-resolved PL decay and fitting of (HMTA)_3_Pb_2_Br_7_ crystals (excited using a 365 nm diode laser, probed at 580 nm) at room temperature.

Fig. 2a shows the (HMTA)_3_Pb_2_Br_7_ single crystals under ambient light and ultraviolet (UV) light (365 nm). The colorless crystals emit yellowish-white light or “warm” white under UV excitation with a reasonably good photostability (Fig. S4†), suggesting below-gap broadband emission with a large Stokes shift. As shown in Fig. 2b, (HMTA)_3_Pb_2_Br_7_ can indeed be excited by UV light from 250 to 400 nm and gives a broad emission peak at 580 nm that covers a wide range of the spectrum from 450 to 750 nm with a large FWHM of 158 nm. We performed two emission scans under different excitation wavelengths (350 nm and 380 nm) to capture the whole emission spectrum. The position and shape of the two emission peaks are almost identical, indicating the excitation-independent emission property of this material. The Commission Internationale de l’Eclairage (CIE) chromaticity coordinates for this yellowish-white emission are calculated to be (0.42, 0.45) (Fig. 2c), which represent a significant red-shift as compared to the bluish-white emission from a bulk assembly of 1D lead bromide nanowires (C_4_N_2_H_14_PbBr_4_).^21^ The PLQE of this material was measured to be around 7%. This relatively low PLQE, as compared to those of organic lead bromide hybrids with 3D, 2D-layered, and 1D-wire structures, is likely due to more non-radiative pathways present in this nanotube structure. The photoluminescence decay was characterized by time-resolved photoluminescence spectroscopy. As shown in Fig. 2d, bi-exponential fitting of the intensity–time curve gives an average lifetime of approximately 106 ns (the functional formula is specified in the ESI†). Such a strongly Stokes shifted broadband emission with a relatively long lifetime is indeed similar to what has been observed in corrugated-2D and 1D metal halide perovskites.^21^,^25^–^33^ It is therefore reasonable to attribute this non-Gaussian shaped broadband emission to the self-trapped excited states with multiple energy minimums, which are in thermally activated equilibrium at room temperature. Unlike emissions from both free excitons and self-trapped excited states in a bulk assembly of metal halide nanowires (C_4_N_2_H_14_PbBr_4_),^21^ only self-trapped emissions were recorded for (HMTA)_3_Pb_2_Br_7_ (Fig. 2b), suggesting the absence of delocalized excitonic states due to the ultrafast exciton self-trapping in (HMTA)_3_Pb_2_Br_7_ at room temperature. This is not surprising if we compare the structure of 1D lead bromide nanotubes to that of nanowires. The electron coupling and molecular orbital interaction between the metal halide dimers *via* corner-sharing in 1D nanotubes are much weaker than those between the edge sharing metal halide octahedra in 1D nanowires. Therefore, the reduced conjugation in metal halide nanotubes produces more localized electronic states with stronger electron–phonon coupling, favoring the formation of self-trapped excitons.

**Fig. 3:**
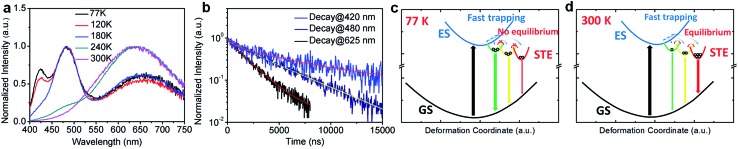
(a) Temperature-dependent PL spectrum of (HMTA)_3_Pb_2_Br_7_ crystals (excited at 380 nm) from 77 K to 300 K. (b) The emission decays at different wavelengths at 77 K and their multi-exponential fitting curves (excited using a 365 nm diode pulse laser, probed at 420 nm, 480 nm and 625 nm). (c–d) Proposed energy diagrams and excited state dynamics of (HMTA)_3_Pb_2_Br_7_ at 77 K and at 300 K: GS = ground state, ES = excited state, STE = self-trapped states, and the straight and curved arrows represent optical and energy relaxation/transfer transitions, respectively. Circles represent self-trapped excitons.

To further confirm the origin of the broadband emission from the self-trapped excited states, we have characterized the temperature-dependent emission property of (HMTA)_3_Pb_2_Br_7_ by measuring its photoluminescence (PL) from 77 K to 300 K (Fig. 3a). At 77 K, three emission peaks at 420 nm, 480 nm and 625 nm were observed. These emissions have different decay lifetimes as shown in Fig. 3b, which are estimated to be 7 μs, 4.0 μs and 1.8 μs from the short wavelength to long wavelength, respectively (the functional formulas are specified in the ESI†). The long emission decay time is consistent with the typically observed slow spin-forbidden triplet exciton emission. Such distinct emission bands with different lifetimes at 77 K clearly indicate that multiple excited states are generated upon photoexcitation, which is consistent with the inherent properties of exciton self-trapping in metal halide perovskites. At low temperature (77 K), the trapped excitons could not overcome the energy barriers between different trapped excited states. In other words, the thermally activated equilibrium is likely suspended. As a result, trapped excited states with higher energy but faster self-trapping processes are kinetically populated and give distinguishable emissions at 420 nm and 480 nm. The formation of these self-trapped excitons with higher energy may involve elongation of the Pb–Br bonds within a PbBr_6_ octahedron.^34^ During the warming process, the peak intensities at 420 nm and 480 nm decreased while the peak intensity at 625 nm increased significantly. This phenomenon indicates that when the temperature rises, the trapped excitons can obtain enough thermal energy to overcome the energy barriers between different trapped states, forming a thermal equilibrium. Therefore, the emission at room temperature is mostly from the self-trapped states which have the lowest energy among all of the excited states. The formation of these trapped states with lower energy may originate fro9m stronger lattice distortion, which requires a larger kinetic barrier.^35^ For clarity, the photoluminescence mechanism for this bulk assembly of metal halide nanotubes is depicted in the configuration coordinate diagram given in Fig. 3c and d. Upon UV excitation, the lead bromide nanotubes are excited to the high energy excited states, which undergo ultrafast exciton self-trapping with the formation of multiple excited states that give multiple emission bands at 77 K without thermally activated equilibrium, and strongly Stokes shifted broadband photoluminescence at room temperature due to thermally activated equilibrium. The observation of multiple exciton emissions with strong temperature dependence resembles the emissions of ns^2^ ions in alkali halides.^36^

**Fig. 4:**
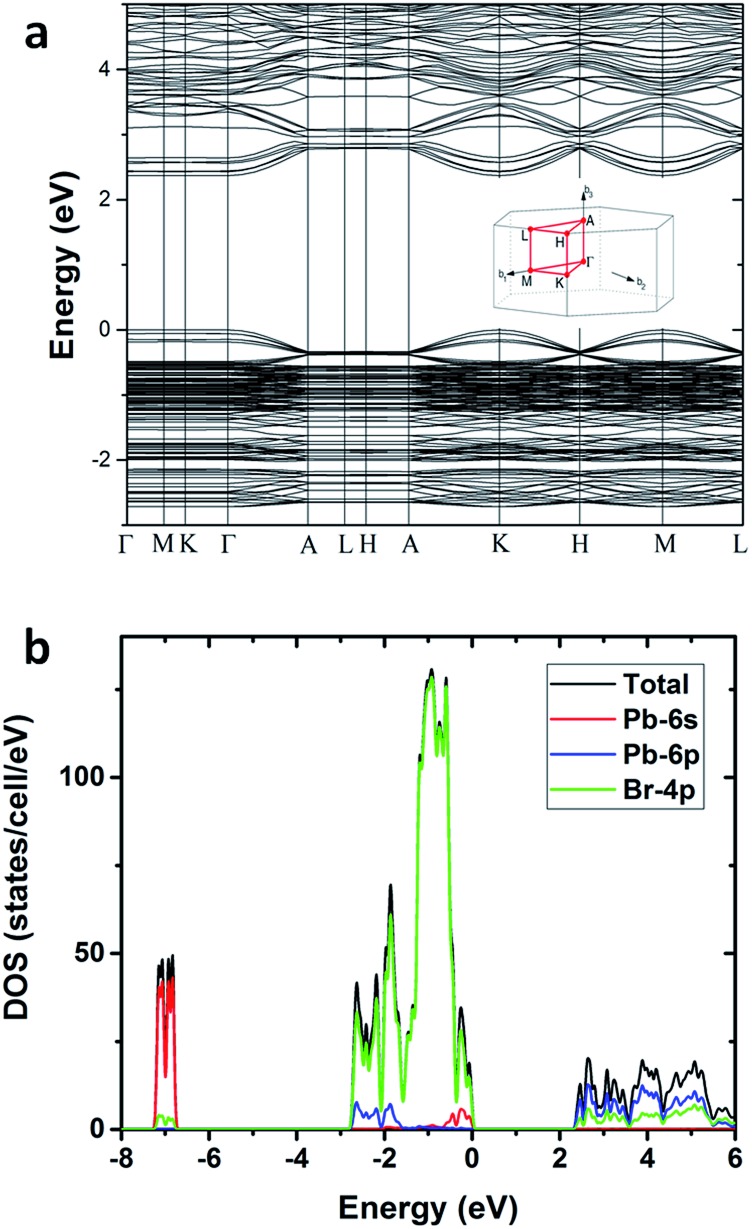
(a) Calculated band structure of (HTMA)_3_Pb_2_Br_7_. (b) Projected density of states of (HTMA)_3_Pb_2_Br_7_.

To gain deeper insight into the physical mechanism underpinning the photophysics of this bulk assembly of metal halide nanotubes, we performed density functional theory (DFT) calculations. The calculated band structure of (HTMA)_3_Pb_2_Br_7_ (Fig. 4a) shows a direct band gap at the *Γ* point. The calculated band gap is 2.37 eV at the Perdew–Burke–Ernzerhof (PBE) level, which is expected to be underestimated due to the well-known band gap error of DFT. The conduction and the valence bands are flat on the plane perpendicular to the axis of the tube, indicating negligible inter-tubular interaction. Along the axis of the tube, the band dispersion is still small, reflecting the weakened Pb–Br hybridization due to the distortion of the PbBr_6_ octahedral structure. The narrow bands near the band edges and the soft lattice of (HTMA)_3_Pb_2_Br_7_ should favor charge localization and the formation of self-trapped excitons,^37^–^39^ which is consistent with the absence of free exciton emission at room temperature. The top of the valence band is mostly made up of Br-4p states but has significant Pb-6s character, while the bottom of the conduction band is dominated by Pb-6p states as shown by the projected density of states (DOS) in Fig. 4b. Thus, excitons should be localized on the Pb^2+^ ions and the emission is likely due to the Pb 6p–6s transition.

## Conclusions

In summary, we have prepared for the first time a novel single crystalline organic metal halide hybrid containing arrays of metal halide nanotubes *via* a simple bottom up solution self-assembly process. Our work bridges the research of organic metal halide hybrids with functional nanotube materials. The excitement of our work lies not only in the specific achievements, but also in what it represents in terms of new opportunities for organic metal halide hybrids beyond conventional structures. On-going research aims to explore the optical, electronic, and other properties of this new class of organic metal halide hybrids with tubular structures, and develop theories to predict and guide the synthesis of possible organic metal halide frameworks. Efforts to improve the photoluminescence properties of such nanotube assemblies by making more rigid structures are also underway.

## Conflicts of interest

There are no conflicts to declare.

## Supplementary Material

Supplementary information

Crystal structure data
