# Supplementary material for: Bulk assembly of organic metal halide nanotubes
Source: Chem Sci. 2017 Oct 16;8(12):8400–4. doi: 10.1039/c7sc03675b (PMC5862099; doi:10.1039/c7sc03675b)
Supplement: Supplementary file 1 [file SC-008-C7SC03675B-s001.pdf]

Electronic Supplementary Material (ESI) for Chemical Science.
This journal is © The Royal Society of Chemistry 2017

Supporting Information

Bulk Assembly of Organic Metal Halide Nanotubes

Haoran Lin,[a] Chenkun Zhou,[a] Yu Tian,[b] Tiglet Besara,[c] Jennifer Neu,[c] Theo Siegrist,[a,b,c] Yan Zhou,[d] James Bullock,[e] Kirk S. Schanze,[e] Wenmei Ming,[f] Mao-Hua Du,[f] Biwu Ma*[a,b,d]

a. Department of Chemical and Biomedical Engineering, FAMU-FSU College of Engineering, Tallahassee, Florida 32310, United States

b. Materials Science and Engineering Program, Florida State University, Tallahassee, Florida 32306, United States

c. National High Magnetic Field Laboratory, Florida State University, Tallahassee, Florida 32310, United States

d. Department of Chemistry and Biochemistry, Florida State University, Tallahassee, Florida 32306, United States

e. Department of Chemistry, University of Florida, Gainesville, Florida 32611, United States

f. Materials Science & Technology Division and Center for Radiation Detection Materials and Systems, Oak Ridge National Laboratory, Oak Ridge, Tennessee 37831, United States

Methods

Materials. Lead (II) bromide, hexamethylenetetramine and hydrobromic acid (48 wt.% in H_2O) were purchased from Sigma-Aldrich. Dichloromethane (DCM, 99.9%) and

dimethylformamide (DMF, 99.8%) were purchased from VWR. All reagents and solvents were used without further purification unless otherwise stated.

Hexamethylenetetramine hydrobromide. Hexamethylenetetramine hydrobromide was prepared by adding hydrobromic acid solution (0.81 mL, 7.1 mmol) into the hexamethylenetetramine (1.00 g, 7.1 mmol) in 150 mL ethanol at 0 °C. The white cotton-like organic salts were obtained (1.1 g, 70 % yield) after removal of the solvents under vacuum, followed by washing with a suitable amount of ethanol. The salts were dried and kept in a desiccator for future use.

Crystal growth of $(C_6H_{13}N_4)_3Pb_2Br_7$ nanotube material. Lead (II) bromide (100 mg, 0.272 mmol) and hexamethylenetetramine hydrobromide (90.0 mg, 0.409 mmol) were mixed at 2:3 molar ratio and dissolved in DMF (5 mL) to form a clear precursor solution. Bulk crystals were prepared by diffusing DCM (2 mL) into the prepared DMF solution (1 mL) at room temperature for overnight. The large colorless crystals were washed with DCM and dried under reduced pressure. The yield was calculated at ~ 53 %. $(C_6H_{13}N_4)_3Pb_2Br_7$: Anal, Calc. C, 15.47; H, 2.81; N, 12.03. Found: C, 15.95; H, 2.92; N, 11.42.

Single crystal X-ray diffraction (SCXRD). Single crystal x-ray diffraction data of hexamethylenetetramine lead bromide [(CH2)6N4]3Pb2Br7 was collected using an Oxford-Diffraction Xcalibur-2 CCD diffractometer with graphite-monochromated Mo Kα radiation. The crystal was mounted in a cryoloop under Paratone-N oil and cooled to 100 K with an Oxford-Diffraction Cryojet. A complete sphere of data was collected using ω scans with 1° frame widths to a resolution of approximately 0.84 Å, equivalent to $2\theta \approx 50°$. Reflections were recorded, indexed and corrected for absorption using the Oxford-Diffraction CrysAlisPro software,[1] and subsequent structure determination and refinement was carried out using the single crystal x-ray structure refinement and analysis software package CRYSTALS,[2] employing Superflip,[3] a program for solution of crystal structures by charge

flipping, to solve the crystal structure. All hydrogens were restrained to the connecting carbons. The refinement was performed against F2, with anisotropic thermal displacement parameters for all non-hydrogen atoms and with isotropic thermal displacement parameters for the hydrogens in the structure. A CIF has been deposited with CCDC (No. 1550500). Three hydrogen atoms in the unit cell are not resolved, which may due to the low signal quality at high reflection angle.

Powder X-ray diffraction (PXRD). The PXRD analysis was performed on Panalytical X'PERT Pro Powder X-Ray Diffractometer using Copper X-ray tube (standard) radiation at a voltage of 40 kV and 40 mA, and X'Celerator RTMS detector. The diffraction pattern was scanned over the angular range of 5-50 degree (2θ) with a step size of 0.02, at room temperature. Simulated powder patterns were calculated by Mercury software using the crystallographic information file (CIF) from single-crystal x-ray experiment.

Excitation spectrum measurements. Excitation spectra of bulk quantum materials were measured at room temperature on a FLS980 spectrofluorometer (Edinburgh Instruments) monitored at maximum of emission spectra.

Photoluminescence and temperature-dependent photoluminescence steady state studies. Steady-state photoluminescence spectra of bulk quantum materials were obtained at room temperature on a FLS980 spectrofluorometer. Measurements of photoluminescence at different temperatures were performed on the crystals sandwiched between glass microscope slides (1 × 2.5 cm). Samples were held inside an OptistatDN variable liquid nitrogen cryostat (Oxford Instruments) at a 45° angle to the excitation source by a 2 cm × 2 cm sample holder fixed to the end of a cryostat sample holder. The emission spectra were collected at 90° to the excitation source with a Photon Technology International (PTI) spectrophotometer with photomultiplier detection system. The monochromatic excitation at λ=380 nm was used. Individual spectra were recorded at intervals of 10 K with the aid of an Omega CYC3200

auto-tuning temperature controller and thermocouple wire affixed to the sample glass slide. Dry nitrogen was passed through the sample compartment to avoid condensation on the outer cryostat windows.

Photoluminescence quantum efficiencies (PLQEs). For photoluminescence quantum efficiency measurement, the samples were excited using light output from a housed 450 W Xe lamp passed through a single grating (1800 l/mm, 250 nm blaze) Czerny-Turner monochromator and finally a 5 nm bandwidth slit. Emission from the sample was passed through a single grating (1800 l/mm, 500 nm blaze) Czerny-Turner monochromator (5 nm bandwidth) and detected by a Peltier-cooled Hamamatsu R928 photomultiplier tube. The absolute quantum efficiencies were acquired using an integrating sphere incorporated into the FLS980 spectrofluorometer. The PLQE was calculated by the equation: $\eta_{QE} = I_S/(E_R - E_S)$, in which I_S represents the luminescence emission spectrum of the sample, E_R is the spectrum of the excitation light from the empty integrated sphere (without the sample), and E_S is the excitation spectrum for exciting the sample. Control samples, rhodamine 101 and blue phosphor $BaMgAl_{10}O_{17}:Eu^{2+}$, were measured using this method to give PLQEs of ~ 98 % and ~ 93 %, which are close to the literature reported values. The PLQEs were double confirmed by a Hamamatsu C9920 system equipped with a xenon lamp, calibrated integrating sphere and model C10027 photonic multi-channel analyzer (PMA). The measurements taking account of indirect PL provided the same results within the error bars.

Time-resolved photoluminescence. Time-Resolved Emission data were collected at room temperature and 77 K (liquid nitrogen was used to cool the samples) using time-correlated single photon counting on a Horiba JY Fluoromax-4 Fluorometer. Samples were excited with 365 nm pulsed diode lasers. Emission counts were monitored at 580 nm at room temperature and at 420, 480, 625 nm separately at 77K. The intensity average lifetime is defined as

$\langle\tau\rangle = \Sigma\, a_i\tau_i^2/\Sigma\, a_i\tau_i$. The multiexponential fitting formulas and average lifetimes are listed as follows:

Room temperature (Figure 2d):

$y = (0.39 \pm 0.06)e^{-x/(42 \pm 4)} + (0.61 \pm 0.06)e^{-x/(123 \pm 7)}$; $\langle\tau\rangle \sim 106\, ns$

77K, peak at 420 nm (Figure 3b):

$y = (0.24 \pm 0.08)e^{-x/(0.8 \pm 0.4)} + (0.57 \pm 0.05)e^{-x/(7 \pm 2)} + (0.07 \pm 0.05)$; $\langle\tau\rangle \sim 7\, \mu s$

77K, peak at 480 nm (Figure 3b):

$y = (0.3 \pm 0.2)e^{-x/(1.6 \pm 0.6)} + (0.7 \pm 0.2)e^{-x/(4.4 \pm 0.8)} + (0.0 \pm 0.01)$; $\langle\tau\rangle \sim 4.0\, \mu s$

77K, peak at 625 nm (Figure 3b):

$y = (0.86 \pm 0.01)e^{-x/(1.83 \pm 0.04)} + (0.09 \pm 0.02)e^{-x/(0.22 \pm 0.07)} + (0.016 \pm 0.003)$; $\langle\tau\rangle \sim 1.8\, \mu s$

Materials photostability study. To test the photostability, a 100 W 20 V mercury short arc lamp was used as continuous irradiation light source. The intensity of the irradiation was calibrated to 150 mW/cm^2. The emission was measured perodically on a HORIBA iHR320 spectrofluorimeter, equipped with a HORIBA Synapse CCD detection system.

Thermogravimetry Analysis (TGA). TGA was carried out using a TA instruments SDT: simultaneous DSC & TGA system of Q600 model. The samples were heated from room temperature (around 22 ^{o}C) to 800 ^{o}C at a rate of 10 ^{o}C·min^{-1}, under an argon flux of 100 mL·min^{-1}.

Calculation. Our calculations were based on density functional theory (DFT) implemented in the plane-wave basis VASP code.[4] The projector augmented wave method was used to describe the interaction between ions and electrons.[5] Experimental lattice constants of hexagonal $(HTMA)_3Pb_2Br_7$ were used: a = 24.5206 Å and c = 9.7661 Å. Since the positions of the protons bonded to the HTMA molecules are not resolved experimentally, we replaced the organic cations by a uniform positive charge background such that the simulation cell is charge neutral. This approximation does not affect the electronic structure near the valence

and the conduction band edges because the electronic states derived from the organic molecules are far from the band gap.[6,7] The kinetic energy cutoff of 216 eV and the 1 × 1 × 2 reciprocal space k-point mesh were used. The atomic positions were fully relaxed until the residual forces were less than 0.02 eV/ Å. The band structure and the density of states (DOS) were calculated using the Perdew−Burke−Ernzerh (PBE) exchange-correlation functional.[8] The spin-orbit coupling (SOC) was included in the calculations.

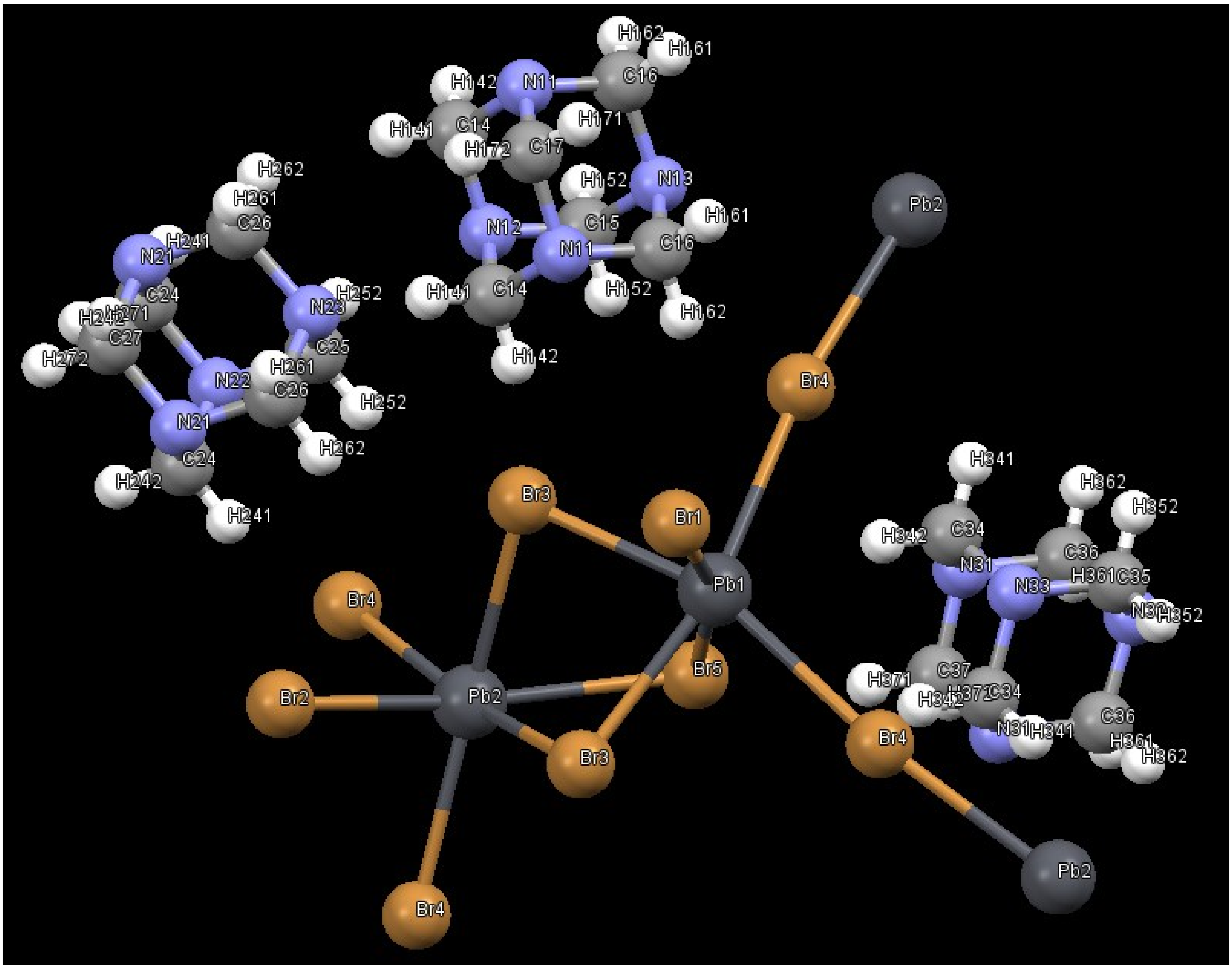

Figure S1. Atomic labels in SCXRD results.

Table S1. Single crystal x-ray diffraction data and collection parameters. The collection was performed at a temperature of 100 K.

Compound	$(HMTA)_3Pb_2Br_7$
Formula	$C_{18}H_{39}N_{12}Pb_2Br_7$
Molecular weight	1394.29 g/mol
Space group	$P6_3/m$ (#176)
a	24.5149(6) Å
c	9.7646(2) Å
V	5082.1(2) Å^3
Z	6
$\rho_{calc.}$	2.733 g/cm^3
μ	18.213 mm^{-1}
Data collection range	2.834° < θ < 25.066°
Reflections collected	69463
Independent reflections	3199
Parameters refined	280
Restraints	108
R_1, wR_2	0.0593^a, 0.0728^b
Goodness-of-fit on F^2	1.0634

$^{a)}$ $R_1 = \Sigma \| F_o | - | F_c \| / \Sigma | F_o |$. $^{b)}$ $wR_2 = [\Sigma w(F_o^2 - F_c^2)^2 / \Sigma w(F_o^2)^2]^{1/2}$.

Table S2. Atomic positions of $(HMTA)_3Pb_2Br_7$. All non-hydrogens were refined with anisotropic displacement parameters, while the hydrogens were refined with isotropic displacement parameters.

Atom	Site	x	y	z	U_{eq} , U_{iso} (Å^2)
Pb1	6h	0.71799	0.65688	0.25	0.023
Pb2	6h	0.65448	0.78652	0.25	0.048
Br1	6h	0.62146	0.52648	0.25	0.023
Br2	6h	0.5182	0.73401	0.25	0.045
Br3	12i	0.64245	0.68525	0.4477	0.029
Br4	12i	0.78504	0.65075	0.51749	0.032
Br5	6h	0.7918	0.80509	0.25	0.038
N11	12i	0.5585	0.4744	0.6254	0.019
N12	6h	0.5814	0.5689	0.75	0.02
N13	6h	0.6569	0.533	0.75	0.025
C14	12i	0.5519	0.5301	0.627	0.017
C15	6h	0.6487	0.59	0.75	0.026
C16	12i	0.6251	0.4941	0.6245	0.027
C17	6h	0.5302	0.437	0.75	0.026
N21	12i	0.458	0.6748	0.6261	0.025
N22	6h	0.5412	0.7645	0.75	0.024
N23	6h	0.536	0.6624	0.75	0.024
C24	12i	0.5001	0.7437	0.6272	0.028
C25	6h	0.5793	0.7339	0.75	0.022
C26	12i	0.4948	0.6443	0.6243	0.025
C27	6h	0.4194	0.6568	0.75	0.028

N31	12i	0.9767	0.8483	0.3739	0.048
N32	6h	1.0524	0.8302	0.25	0.044
N33	6h	0.9414	0.7513	0.25	0.035
C34	12i	0.932	0.7802	0.3724	0.039
C35	6h	1.0041	0.7624	0.25	0.039
C36	12i	1.0407	0.8578	0.3732	0.048
C37	6h	0.9703	0.8783	0.25	0.052
H142	12i	0.5706	0.5544	0.544	0.022
H141	12i	0.5075	0.5167	0.628	0.022
H152	12i	0.6681	0.615	0.8313	0.034
H162	12i	0.6441	0.5186	0.541	0.035
H161	12i	0.6298	0.4572	0.625	0.035
H171	6h	0.537	0.4015	0.75	0.033
H172	6h	0.4853	0.422	0.75	0.033
H242	12i	0.4748	0.764	0.627	0.035
H241	12i	0.5257	0.7558	0.544	0.035
H252	12i	0.6056	0.7464	0.8312	0.028
H262	12i	0.5208	0.6572	0.541	0.031
H261	12i	0.4671	0.5992	0.624	0.031
H272	6h	0.3942	0.677	0.75	0.037
H271	6h	0.392	0.6116	0.75	0.037
H341	12i	0.939	0.7617	0.455	0.049
H342	12i	0.8895	0.7732	0.373	0.049
H352	12i	1.0097	0.7431	0.3313	0.048
H361	12i	1.0707	0.9025	0.375	0.06
H362	12i	1.0462	0.839	0.456	0.06
H372	6h	1.002	0.9223	0.25	0.067
H371	6h	0.929	0.874	0.25	0.067

Table S3. Pb-Br bond lengths in $(HMTA)_3Pb_2Br_7$.

Bond	Distance (Å)
Pb1 – Br1	2.873
Pb1 – Br3	2.988
Pb1 – Br4	3.129
Pb1 – Br5	3.147
Pb2 – Br2	2.919
Pb2 – Br3	3.041
Pb2 – Br4	3.018
Pb2 – Br5	3.163

Table S3. Br-Pb-Br bond angles in $(HMTA)_3Pb_2Br_7$.

Bond angle	Degree (°)
Br1 – Pb1 – Br3	89.85
Br1 – Pb1 – Br4	96.13
Br3 – Pb1 – Br3	80.49
Br3 – Pb1 – Br4	82.73

Br3 – Pb1 – Br5	78.27
Br4 – Pb1 – Br4	113.15
Br4 – Pb1 – Br5	92.46
Br2 – Pb2 – Br3	91.01
Br2 – Pb2 – Br4	83.54
Br3 – Pb2 – Br3	78.83
Br3 – Pb2 – Br4	91.48
Br3 – Pb2 – Br5	77.25
Br4 – Pb2 – Br4	97.57
Br4 – Pb2 – Br5	106.24

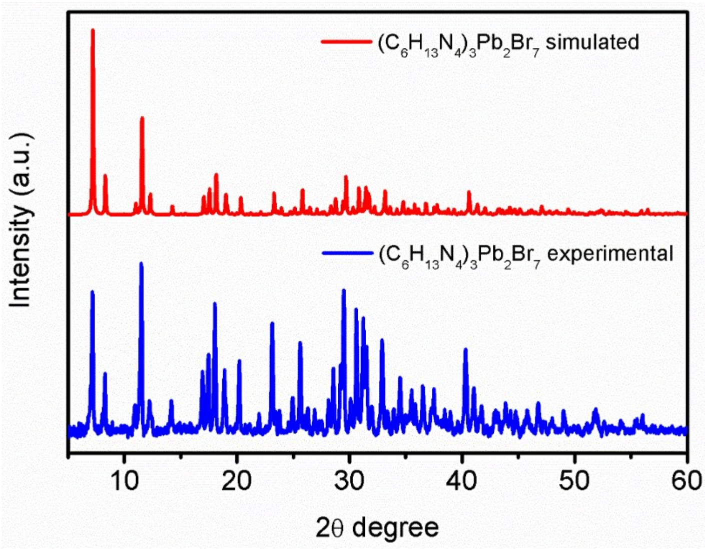

Figure S2. PXRD data of $(HTMA)_3Pb_2Br_7$ bulk quantum material as well as the simulated results from the SCXRD data.

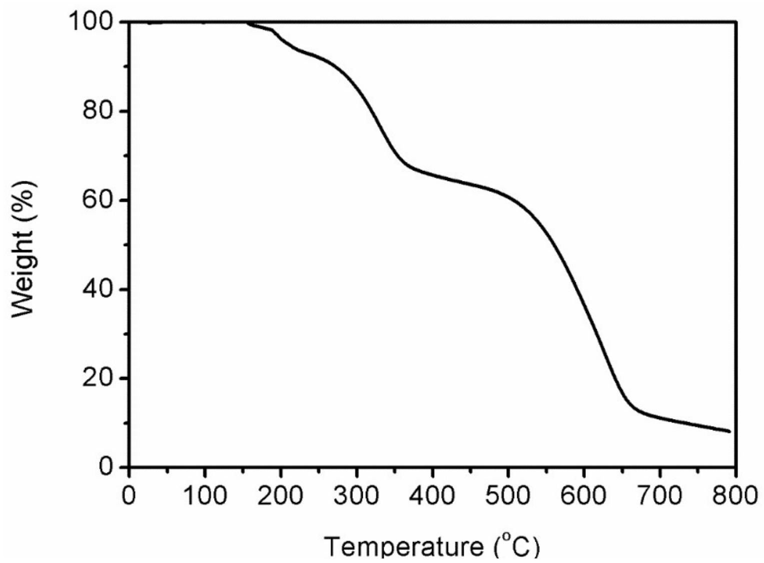

Figure S3. TGA data of $(HTMA)_3Pb_2Br_7$ bulk quantum material.

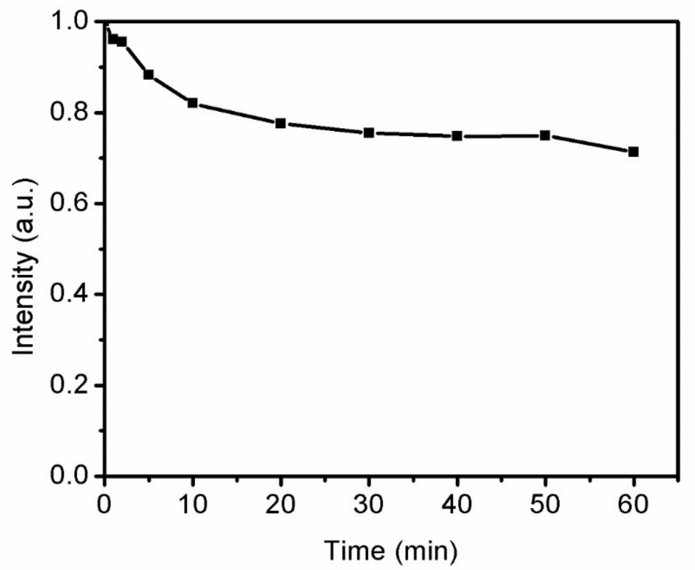

Figure S4. Photostability of $(HTMA)_3Pb_2Br_7$ bulk quantum material under continuous illumination using a high power mercury lamp (150 mW/cm^2).

References

1 Rigaku Oxford Diffraction, 2017, CrysAlisPro Software system, version 1.171.38.43, Rigaku Corporation, Oxford, UK.

2 P. W. Betteridge, J. R. Carruthers, R. I. Cooper, K. Prout and D. J. Watkin, *J. Appl. Cryst.* 2003, **36**, 1487. We used version 14 build 6236 (March 2017).

3 L. Palatinus and G. Chapuis, *J. Appl. Cryst.* 2007, **40**, 786-790.

4 G. Kresse and J. Furthmüller, *Comput. Mater. Sci.* 1996, **6**, 15-50.

5 G. Kresse and D. Joubert, *Phys. Rev. B* 1999, **59**, 1758-1775.

6 M. H. Du, *J. Mater. Chem. A* 2014, **2**, 9091-9098.

7 T. Umebayashi, K. Asai, T. Kondo and A. Nakao, *Phys. Rev. B* 2003, **67**, 155405.

8 J. P. Perdew, K. Burke and M. Ernzerhof, *Phys. Rev. Lett.* 1996, **77**, 3865-3868.
